# The long sepsis journey in low- and middle-income countries begins with a first step...but on which road?

**DOI:** 10.1186/s13054-018-1987-z

**Published:** 2018-03-09

**Authors:** Arthur Kwizera, Inipavudu Baelani, Mervyn Mer, Niranjan Kissoon, Marcus J. Schultz, Andrew J. Patterson, Ndidiamaka Musa, Joseph Christopher Farmer, Martin W. Dünser, Sepsis in Resource-Limited Settings Workgroup of the Surviving Sepsis Campaign, Neil Adhikari, Pedro R. Arriaga, Marie R. Baldisseri, Satish Bhagwanjee, Daniel H. Ceraso, Daniel H. Ceraso, Andrew P. L. Clift, Lori A. Harmon, Judith Hellman, Jorge L. Hidalgo, Halima S. Kabara, Ruth M. Kleinpell, Asad Latif, Ganbold Lundeg, Bhavesh Patel, Nestor O. Raimondi, Narendra Rungta, Gentle S. Shrestha, Jose M. Teles, Guillaume Thiery, Janice L. Zimmerman

**Affiliations:** 1 Intensive Care Unit, Department of Anaesthesia, Makerere University College of Health Sciences and Mulago National Referral Hospital, Kampala, Uganda; 2 Department of Anesthesia, Doctors On Call for Service (DOCS) Education Hospital, Goma, Democratic Republic of the Congo; 3 Intensive Care Unit, Charlotte Maxeke Johannesburg Academic Hospital and University of the Witwatersrand, Johannesburg, South Africa; 4 BC Children’s Hospital, University of British Columbia, Vancouver, Canada; 5 Academic Medical Center, University of Amsterdam, Amsterdam, The Netherlands; 6 Department of Anesthesiology, University of Nebraska Medical Center, Omaha, USA; 7 Seattle Children’s Hospital, University of Washington, Seattle, USA; 8 Department of Critical Care Medicine, Mayo Clinic, Phoenix, USA; 9 Department of Anesthesiology and Intensive Care Medicine, Kepler University Hospital, Johannes Kepler University Linz, Linz, Austria

**Keywords:** Sepsis, Low-income countries, Middle-income countries, Infection, Prevention, Organ dysfunction

## ᅟ

*“The most efficacious critical care is the critical care intervention that a patient never needs.”* JC Farmer [1].

*“A leader is best when people barely know he exists. When his work is done, his aim fulfilled, they will say: we did it ourselves.”* Lao Tzu [2].

**Fig. 1:**
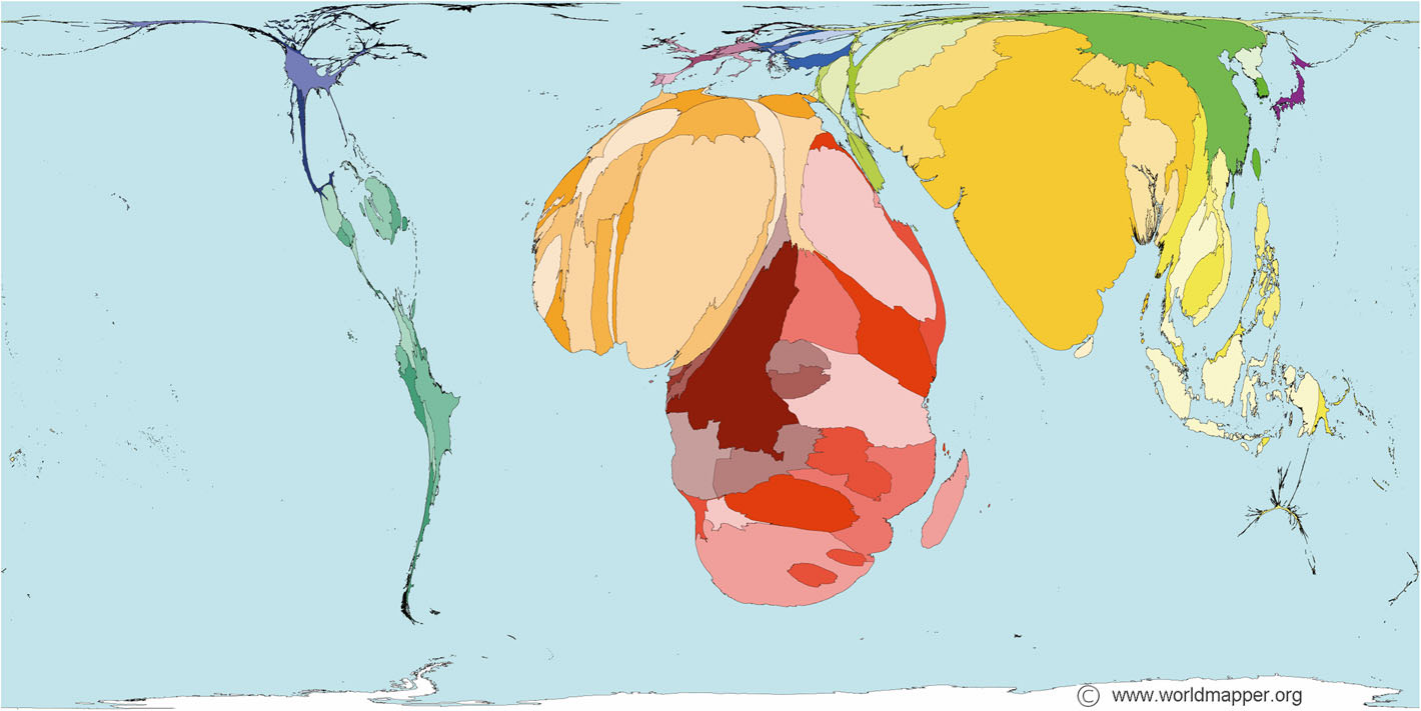
Infectious and parasitic deaths worldwide. Based on data from 2002 including International Classification of Diseases-10 codes: A00-B99, G00, G03-G04, N70-N73. © Copyright Worldmapper.org/Sasi Group (University of Sheffield) and Mark Newman (University of Michigan)

Infection and sepsis [3] are among the most frequent acute medical conditions worldwide and result in approximately eight million premature deaths each year, most of which occur in low and lower-middle income countries (LMICs) (Fig. 1) [4]. The United Nations World Health Assembly has recognized sepsis as a global health priority and adopted a resolution to improve its worldwide prevention, diagnosis and management [5]. Despite the rising global awareness, there are no successful approaches to reduce the sepsis burden in LMICs. This is exacerbated by the reality that contemporary scientific evidence and guidelines on sepsis management almost exclusively originate in high-income countries (HICs). International sepsis guidelines focus on critical care aspects of bacterial or fungal sepsis [6, 7] and cannot be extrapolated to resource-limited settings or patients with non-bacterial sepsis such as malaria or tropical viral diseases. Furthermore, implementation of international guidelines in LMICs may even have harmful effects [8, 9], particularly in vulnerable populations such as children [10]. Strategies specifically designed to reduce the burden of sepsis in LMICs are therefore greatly needed. Solutions must be simple, easily applicable, reliant on frugal and ubiquitous technology, and cost-effective.

Delayed or inadequately treated infection accounts for a relevant number of patients with sepsis in LMIC hospitals. This implies that early and appropriate management of patients with acute infection could reduce the incidence of sepsis-associated morbidity and mortality. Such an approach may conserve already scarce resources where intensive care expertise and required technologies to care for critically ill patients (with sepsis) are widely unavailable or only accessible at excessive costs. In striking contrast to current strategies to reduce sepsis deaths in high-income countries, such a “preventive” approach would paradoxically not focus on critically ill patients but interrupt the pathway from acute infection to sepsis and hence avert organ dysfunction. Following early recognition of at-risk patients, appropriate infection management would focus on timely and adequate antimicrobial therapy as well as surgical source control. All of these interventions should be simple, also available in non-physician-staffed primary care facilities (e.g. dispensaries, health care centres) and not depend on technology or sophisticated interventions.

Sepsis “prevention” in LMICs is an incomplete approach. Some patients either present late in their disease course or have sepsis that is pre-determined by the type and severity of infection (e.g. peritonitis), pre-morbid conditions or individual genotypes. Thus, a strategy to reduce sepsis mortality in LMICs should also include a rational treatment plan for patients with infection-induced organ dysfunction. Achievable interventions (in addition to adequate source control) may include intravenous glucose administration, early and judicious fluid administration, selective oxygen application and (if available) non-invasive ventilatory strategies. If available, intensive care facilities should also be accessible to critically ill patients with sepsis. However, sound triage principles dictate that resources should not be diverted from the care of non-critically ill patients with acute infection to interventions for those patients with organ dysfunction and an exponentially increased risk of death.

Despite the obvious simplicity of this suggested approach, many barriers may prevent its widespread adoption and implementation. Unlike in high-income countries where the focus of initiatives to improve sepsis care rely on the presence of emergency and intensive care resources, medical care providers in primary or secondary level health facilities (initial care) rather than critical care specialists will be the architects of this new approach. Awareness and knowledge regarding the burden of acute infection and time-sensitivity of its treatment among these healthcare professionals may be limited. Furthermore, antimicrobial resistance is widespread in LMICs, rendering commonly available antimicrobials ineffective [11].

To overcome these barriers and to successfully implement an approach that optimizes the management of patients with acute infection, we developed a “SCAN-TEACH-TREAT” program. The SCAN component refers to the initial evaluation of the local epidemiology of acute infections, antimicrobial resistance, as well as capacities, human and material resources of health care facilities in the region of interest. Public health considerations are included in the SCAN as well. The findings of the SCAN component are then used to direct the content of the subsequent TEACH component. Concise and deliberate educational interventions on early recognition and management of patients with acute infections should be directed towards hospital- and community-based medical personnel, including physicians, nurses and students. In parallel, advocacy efforts must be launched to raise awareness about the burden of infection and sepsis among political stakeholders and the public. Finally, the third component of the approach is the TREAT module, which includes the implementation of pragmatic and simple infection treatment bundles into practice. A ready-to use “Sepsis First Aid” kit (e.g. in the form of a carton box, plastic bag or cart) containing key resources to implement these bundles (e.g. a choice of antimicrobials adjusted to the regional antimicrobial resistance pattern) may optimize patient care, particularly in regions facing severe resource restrictions.

The overarching goal of such a program is to reduce the risk and impact of sepsis in LMICs. Even when a program demonstrates benefit and cost effectiveness, it will not succeed without the strong support and endorsement of local health and governmental leaders. Indeed, the most important success factor is seamless cooperation between program developers and local/regional participants. Hence, it is essential that local medical professionals and governmental leaders actively direct and execute the program.

In summary, the outcome effects of clinical protocols like SCAN-TEACH-TREAT must be closely monitored and rigorously evaluated. Additionally, we must assess treatment effects for LMIC sepsis patients with established organ dysfunction who receive (resource-limited) ICU interventions. Impact and benefit must be measured. Finally, scholarly assessment and clinical publications are valuable success-building tools that do not only increase research capacities, but also foster clinical leadership in LMICs. In addition to academic merits, these can also serve as documentation/motivation to government leaders that the people they govern receive measurable health and welfare benefits from the programs that they support.

## Abbreviations

ICU: Intensive care unit
LMIC: Low- and lower-middle income country
SSC: Surviving Sepsis Campaign
